# Crystalline cataract caused by a heterozygous missense mutation in γD-crystallin (*CRYGD*)

**Published:** 2011-12-20

**Authors:** Deborah K. VanderVeen, Caroline Andrews, Bharti R. Nihalani, Elizabeth C. Engle

**Affiliations:** 1 Department of Ophthalmology, Children's Hospital Boston, Harvard Medical School, Boston MA; 2 Department of Neurology, M Kirby Neurobiology Center, and The Manton Center for Orphan Disease Research, Children’s Hospital Boston, Harvard Medical School, Boston, MA; 3 Howard Hughes Medical Institute, Chevy Chase MD

## Abstract

**Purpose:**

To describe phenotypic characteristics of two pedigrees manifesting early onset crystalline cataract with mutations in the γD-crystallin gene (*CRYGD*).

**Methods:**

A detailed medical history was obtained from two Caucasian pedigrees manifesting autosomal dominant congenital cataracts. Genomic DNA was extracted from saliva (DNA Genotek). Single Nucleotide Polymorphism (SNP) based genome analysis of the larger pedigree revealed linkage to an 8.2 MB region on chromosome 2q33-q35 which encompassed the crystallin-gamma gene cluster (*CRYG*). Exons and flanking introns of *CRYGA*, *CRYGB*, *CRYGC* and *CRYGD* were amplified and sequenced to identify disease-causing mutations.

**Results:**

A morphologically unique cataract with extensive refractile “crystals” scattered throughout the nucleus and perinuclear cortex was found in the probands from both pedigrees. A heterozygous C→A mutation was identified at position 109 of the coding sequence (R36S of the processed protein) in exon 2 of *CRYGD* and this missense mutation was found to cosegregate with the disease in the larger family; this mutation was then identified in affected individuals of pedigree 2 as well.

**Conclusions:**

The heterozygous 109C→A *CRYGD* missense mutation is associated with a distinct crystalline cataract in two US Caucasian pedigrees. This confirms crystalline cataract formation with this mutation, as previously reported in sporadic childhood case from the Czech Republic and in members of a Chinese family.

## Introduction

Congenital and developmental cataracts are an important cause of visual impairment in children [1]. Most bilateral congenital cataracts, whether familial or sporadic, consist of nuclear opacities. Many of these nuclear opacities are dense at birth and require early surgery, while others are progressive [2]. Several phenotypically unique morphologic variants have been previously described, and are easily recognizable by the examining ophthalmologist. Examples include the cerulean cataract [3], the pulverulant and/or zonular type congenital cataracts (Coppock-type) [4], the aculeiform cataract [5,6], and the coralliform cataract [7-9]. Most of these cataracts consist of whitish appearing opacities, and lens changes that appear “crystalline” are uncommon.

Studies of families with heritable cataract commonly show mutations in genes for lens crystallins. Crystallins (α-, β-, γ-) are the major water soluble proteins expressed in the lens, and play a critical role in maintaining lens clarity. Mutations in the γ-crystallin gene (most commonly *CRYGC* and *CRYGD*) are responsible for multiple types of autosomal dominant cataracts, including pulverulent, aceuliform, cerulean, and lamellar cataracts [2]. Correlations have been noted between specific missense mutations in the γ-crystallin genes and specific cataract phenotypes.

Previously, a 109C→A missense mutation in *CRYGD* was identified in a 5-year-old Czech boy [10] with a unique crystalline cataract, and in a Chinese family with congenital “golden” crystalline lens changes [11]. In this report we describe a crystalline cataract with the recognizable feature of clear refractile crystal formation in two families, and find that both harbor the *CRYGD* 109C→A missense mutation.

## Methods

Members of two families manifesting autosomal dominant congenital cataracts participated in this genetic study, approved by the Institutional Review Board of Children’s Hospital, Boston, MA. Signed informed consent was obtained from all participants or their guardians, conforming to the Declaration of Helsinki. The probands presented with congenital cataracts and underwent detailed ophthalmologic evaluations. Clinical characteristics of the probands were documented, and family ocular history was obtained. Medical records with regard to cataract description in family members were not available, and all affected family members had already undergone bilateral cataract removal at the time of this study.

For each study participant, genomic DNA was extracted from saliva and saliva sponge kits using the purifier solution (DNA Genotek Inc., Kanata, Ontario, Canada). Genome-wide Single Nucleotide Polymorphism (SNP) data for linkage analysis was obtained from participating members of pedigree 1 using the Affymetrix’s GeneChip Human Mapping 10K 2.0 SNP array (Affymetrix, Santa Clara, CA). The resulting CHP files were imported into Progeny Software, cleaned, and then exported for linkage analysis. Fast multipoint linkage analysis was performed using Allegro version 2 assuming a dominant mode of inheritance with full penetrance and a disease gene frequency of 0.0001 [12]. Based on the linkage results presented below, the four genes in the crystalline-gamma gene cluster (*CRYGA, CRYGB*, *CRYGC*, and *CRYGD*) were positional gene candidates. Primer sets comprising exonic and flanking intronic sequences of these genes were designed based on the NCBI36/hg18 human reference assembly. DNA from participants of both pedigrees were amplified using HotStar Taq DNA polymerase (Qiagen, Hilden, Germany) and directly sequenced by Sanger sequencing (ABI Sequence Analyzer; Applied Biosystems, Foster City, CA). Sequencher (Gene Codes Corporation, Ann Arbor, MI) was then used to analyze the sequence data.

## Results

### Clinical description of probands

A female infant member of Pedigree 1 was first examined in the Ophthalmology clinic at 5 weeks of age. Bilateral cataracts had been suspected on prenatal ultrasound, and her pediatrician confirmed a poor red reflex after birth. She had also been diagnosed prenatally with a complex congenital heart defect with transposition of the great arteries, for which she remained hospitalized after birth and underwent repair at one week of age. There was an extensive family ocular history of congenital cataract, however no other family member had congenital heart disease or other congenital systemic abnormalities.

Complete ophthalmoscopic exam was performed. She was noted to fix on lights, without following; pupillary reactions were normal. Intraocular pressures were 8 mmHg by Perkins tonometry. Even after dilation, a poor red reflex was seen, and retinoscopy could not be performed. The patient underwent uneventful sequential cataract extraction at 8 and 9 weeks of age in the right and left eye, respectively. She was left aphakic and fitted with contact lens correction. She eventually underwent intraocular lens implantation and her Snellen visual acuity at age 10 years was 20/50 in each eye.

A male infant member of Pedigree 2 was seen for ophthalmologic evaluation at 1 week of age, after his pediatrician detected an abnormal red reflex in the newborn nursery. Tiny “specks” were seen in the nucleus of the lens, with clear peripheral and intervening lens, allowing a clear view to the fundus using direct ophthalmoscopy and retinoscopy. He was re-examined every 3 months to monitor the density of the cataract and its impact on visual acuity. By 4 years of age, the density of the specks, which now appeared crystal-like, increased, with a corresponding decline in visual acuity to 20/125 in the right eye and 20/200 in the left eye. He underwent cataract extraction in both eyes with intraocular lens implantation. The final corrected Snellen visual acuity was 20/20 for each eye.

### Morphology of cataracts

**Figure 1:**
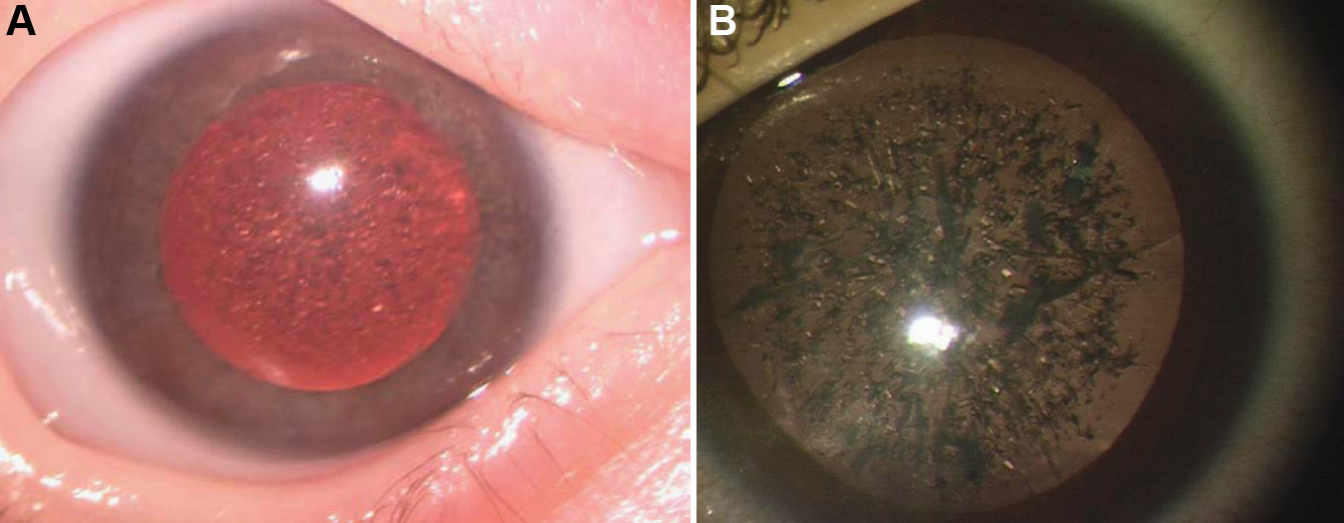
Cataract morphology. **A**: proband of Pedigree 1 and **B**: proband of Pedigree 2 as viewed under an operating microscope.

The cataracts of both probands were morphologically unique from more common types of congenital nuclear cataracts, with extensive refractile “crystals” scattered throughout the nucleus and perinuclear cortex. Intervening areas of clear lens could be seen. Under the operating microscope these crystals were confirmed to be essentially transparent, but were densely packed and refractile (Figure 1). No whitish or gray opacities in the crystals were seen. As evidenced by the patient from pedigree 2, progression of crystal formation was noted both by a decline in vision and by inability to perform retinoscopy or adequately view the fundus.

### Description of pedigrees

**Figure 2:**
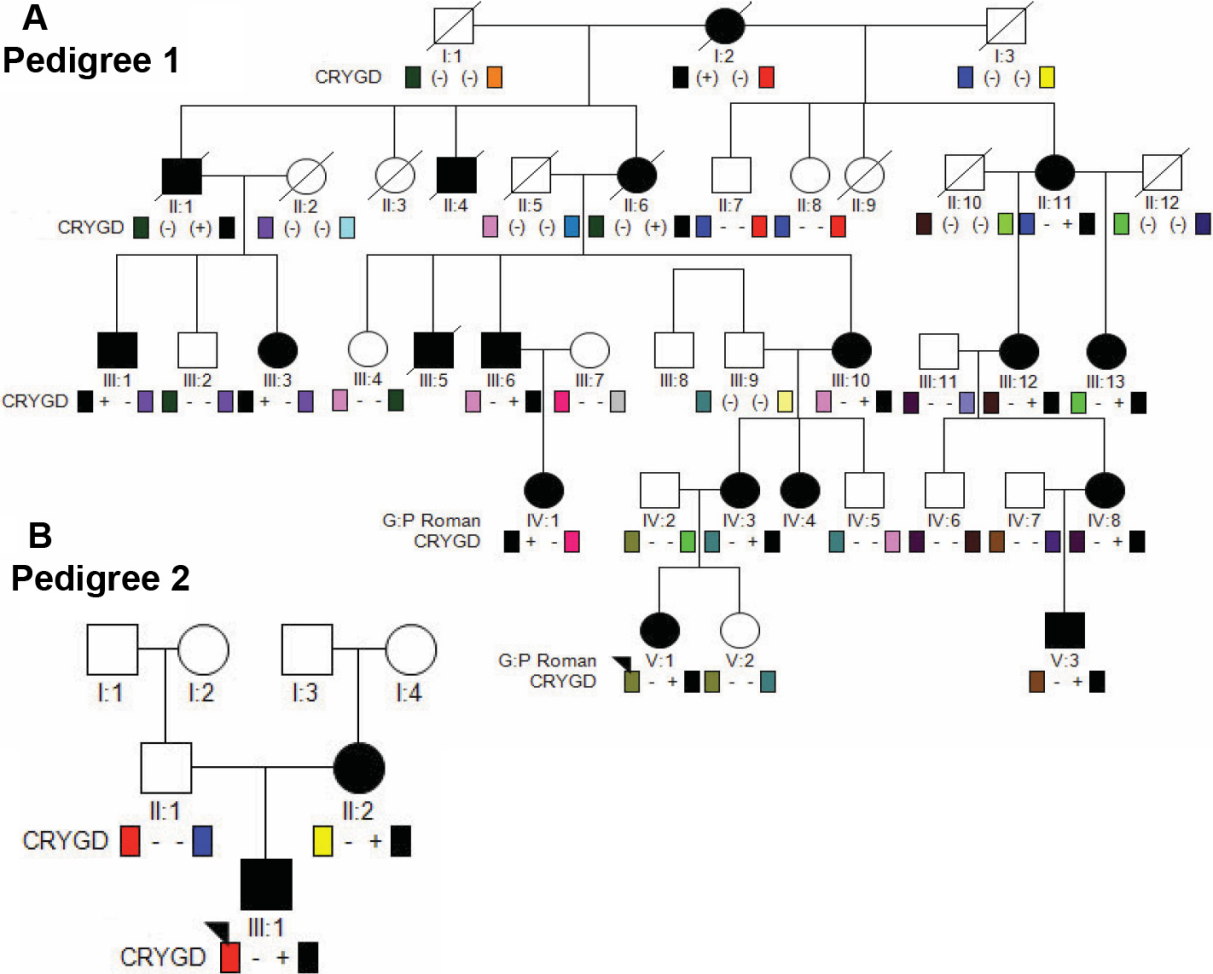
Schematic representation of pedigrees. For each pedigree, squares and circles denote males and females, respectively. Filled squares and circles denote that the individual had cataracts. Diagonal line denotes deceased family member. The proband for each pedigree is indicated by an arrowhead. **A**: Pedigree 1. For each participant, ‘+’ indicates presence of the *CRYGD* 109 C→A mutation (R36S) and ‘-‘ indicates a wildtype allele. Note that the heterozygous *CRYGD* mutation segregates with the dominant crystalline cataract phenotype. **B**: Pedigree 2. The affected mother and son harbor a heterozgous *CRYGD* 109 C→A mutation, while the unaffected father has two wildtype alleles.

Both pedigrees segregate early onset cataract in an autosomal dominant fashion. Pedigree 1 is a five-generation Caucasian (Northern European) pedigree from which 23 individuals were enrolled, 12 of whom were affected (Figure 2A). The family history was reviewed and records obtained for this child’s mother, who had cataract removal at 2 years of age but was left with deprivation amblyopia due to late intervention. All affected family members were either aphakic or pseudophakic, though timing and technique of cataract removal varied by historical standard of care. Pedigree 2 is a 2-generation Caucasian Iranian pedigree from which 3 individuals were enrolled, 2 of whom were affected (Figure 2B). All affected family members also had bilateral involvement, with cataract removal performed after detection per historical standard of care.

### Genetic analysis

Genetic analysis of pedigree 1 revealed linkage to an 8.2 MB region on chromosome 2q33-q35 with a maximal Log of Odds (LOD) score of 4.15. The linked region encompassed the crystallin-gamma gene cluster (*CRYG*).

**Figure 3:**
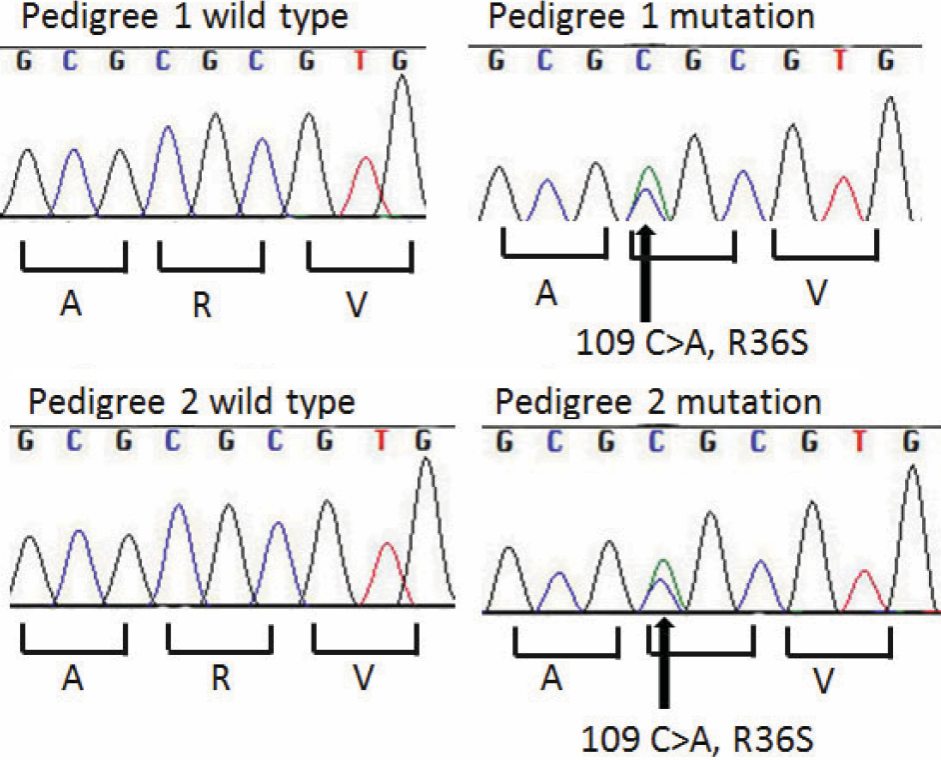
Chromatogram showing sequence analysis of *CRYGD* at exon 2. Chromatograph of an affected (V:1) and unaffected (V:2) individual from Pedigree 1 and unaffected (III:1) and unaffected (II:1) individual from Pedigree 2 in in which a C→A transversion at position 109 resulted in the R36S mutation.

The four genes in *CRYG* cluster were sequenced. No mutations were identified in *CRYGA, CRYGB,* or *CRYGC*. A heterozygous C→A transversion was identified at position 109 in exon 2 of *CRYGD* (109C→A) in each of the probands, and co-segregated with the disease phenotype in both pedigrees (Figure 3). This nucleotide substitution is predicted to result in the substitution of the wild type positively charged and bulky arginine residue for a polar uncharged serine residue (R36S of the processed, NH_2_-terminal methionine-lacking CRYGD protein) [10]. The arginine residue is highly conserved. Kmoch et al. [10] and Gu et al. [11] previously reported absence of the mutation in 200 control alleles and it is not reported in dbSNP build 132.

## Discussion

*CRYGC* and *CRYGD* encode abundant lens γ-crystallins in humans, and almost 90% of γ-crystallins synthesized in human lens are the products of these two genes [13,14]. *CRYGD* γ-crystallins are expressed at high concentrations in the fiber cells of the human embryonic lens and these cells subsequently form the lens nucleus fibers. Mutations in *CRYGC* and *CRYGD* have been identified to cause autosomal dominant congenital cataract as a result of altered stability, association and/or solubility of γ-crystallins [15-22]. We report 2 Caucasian families with a phenotypically distinct early onset crystalline cataract associated with a heterozygous 109C→A missense mutation in *CRYGD* that is predicted to result in a R36S amino acid substitution. The density of the crystals, along with the refractile diffraction and prismatic effects, was the cause of visual morbidity, rather than the opaque whitish changes described in other types of congenital crystalline cataracts, including the aculeiform and coralliform crystalline cataracts.

There have been two previous reports of congenital cataracts in patients harboring the identical 109C→A point mutation in *CRYGD*. The cataract phenotype in both of the current probands was most similar to that in the first report of a sporadic case described by Kmoch et al. [10]. This 5-year-old boy had bilateral cataracts with symmetric crystal deposition and greyish opacities in both lenses. In both lenses numerous birefringent pleiochromic, macroscopically prismatic crystals were seen, with a higher density in the fetal and infantile nucleus and a lower density in the lens cortex. Using protein crystallography, the authors demonstrated that the R36S amino acid substitution alters molecular surface charges and decreases protein solubility, resulting in crystal formation. In the second report, Gu et al. [11] described a pedigree that co-segregated the R36S *CRYGD* amino acid substitution with slowly progressing bilateral nuclear cataracts consisting of a “central pulverulent opacity affecting the embryonal, fetal, and infantile nucleus of the lens, characterized by golden crystal punctuate, with metal-like refractivity in the opaque nucleus.”

The difference in phenotype among pedigrees harboring the identical 109C→A *CRYGD* heterozygous missense mutation (prismatic effects versus metal like or grayish opacities) may be related to the effect of aging. It is interesting to note that the probands in our study presented at a young age when the lens is expected to be clear, and hence, only prismatic and refractile effects were seen. It is possible that the nucleus becomes more brunescent and other opacities may develop with the effect of aging, and may contribute to the “metal like” color or gray opacities as observed in the Chinese family. Alternatively, it is possible that the R36S phenotype is modified by genetics or the environment, as has been demonstrated for the R58H CRYGD substitution that also results in a crystalline cataract. This substitution typically causes the aculeiform crystalline cataract, a phenotypically recognizable cataract characterized by white fiberglass-like or needle-like crystals projecting in different directions [23]. In one four-generation Mexican family segregating the R58H CRYGD substitution, however, all mutation-positive family members had congenital aculeiform cataracts with the exception of one, who had a “coral-like” cataract that is a phenotype classically distinguished from the aculeiform cataract [24]. Pande et al. [25] has demonstrated that both the R36S and R58S CRYGD mutant proteins are much less soluble and more prone to crystallization than the wild type human gamma-B-crystallin protein, but the pathophysiological cause of the phenotypic variability is not yet understood.

Variable onset or rate of progression of cataract is also known to occur, as reported by Stephan et al. [18] in a 3-generation family with punctate progressive juvenile cataract associated with R14C mutation in *CRYGD*. In this family, the lenses were clear at birth, but developed focal greyish-white punctate opacities in the nucleus and surrounding deep cortex with time that required lens extraction. Protein modeling suggested that the effect of this mutation was a subtle one, affecting the surface properties of the crystallin molecule rather than its tertiary structure, consistent with the fact that the lenses were normal at birth. Nandrot et al. [26] found a mutation in *CRYGD* (P23T) of chromosome 2q33–35 in a 4-generation Moroccan family with autosomal dominant cerulean cataract. This mutation alters the protein folding or decreases the thermodynamic stability or solubility of the protein. A different mutation in the same codon (P23S) was found by Plotnikova et al. [27] to be the basis of the polymorphic congenital cataract reported by Rogaev et al. [28]. McManus et al. [29] found that CRYGD proteins with cataract-associated mutations of pro23 became less soluble as temperature increased, in dramatic contrast to the native protein.

Crystalline lens opacities have also been described as a phenotypic variant of the progressive childhood cataract seen in the Hyperferritinemia Cataract Syndrome, but in this condition the crystals appear as square solid opacities, with the clinical appearance usually described as pulverulent or sunflower like [30-33]. These cataracts are caused by mutations in the ferritin light chain (FTL) on chromosome 19. Individuals with this syndrome have hyperferritinemia, and elevated ferritin levels in extracted lenses have also been noted [34].

In conclusion, we confirm that an autosomal dominant crystalline cataract, recognizable by distinct phenotypic features, is associated with an R36S *CRYGD* crystallin mutation in 2 pedigrees described in this article. This cataract is congenital and progressive, so that surgery was performed eventually for all affected family members, with both probands operated in early childhood.
